# Massive Valproic Acid Overdose With Confirmed Pharmacobezoar Formation and Neuromuscular Toxicity: A Case Report and Literature Review

**DOI:** 10.7759/cureus.96884

**Published:** 2025-11-15

**Authors:** Maryam K Alrazooqi, Lara Abumuaileq, Sania Zia, Sara Kazim

**Affiliations:** 1 Emergency Medicine, Rashid Hospital Trauma Center, Dubai, ARE

**Keywords:** continuous renal replacement therapy, extracorporeal therapy, hyperammonemia, l-carnitine, neuromuscular toxicity, pharmacobezoar, valproic acid overdose

## Abstract

We present a 42-year-old man who arrived obtunded to our emergency department (ED) after ingesting 60 g of extended-release valproic acid (VPA). Imaging and endoscopy confirmed a pharmacobezoar, a rare complication of overdose.The patient was managed with early airway protection, gastrointestinal (GI) decontamination, intravenous (IV) levocarnitine (L-carnitine), naloxone, and continuous renal replacement therapy (CRRT). His initial hospital course was stable but later complicated by delayed neuromuscular toxicity with extrapyramidal symptoms (EPS) requiring inpatient rehabilitation and multidisciplinary follow-up.

This case highlights the clinical challenge of massive VPA overdose complicated by pharmacobezoar formation and delayed neuromuscular sequelae. Early risk assessment led to recognition and aggressive treatment of a potentially life-threatening ingestion in which airway protection, serial monitoring, endoscopic confirmation, and extracorporeal elimination were crucial to a favorable outcome. This case underscores the need for vigilance in severe VPA ingestions and reinforces the role of advanced diagnostic and therapeutic strategies in preventing further deterioration.

## Introduction

Valproic acid (VPA), a branched short-chain fatty acid first synthesized in 1882, was originally used as an inert organic solvent [1]. Its anticonvulsant properties were discovered incidentally in 1963, when it was found to suppress chemically induced seizures in rodents [1]. Since then, VPA has become a widely used antiepileptic and mood-stabilizing agent, also indicated for migraine prophylaxis [2]. Its therapeutic effects are primarily mediated through enhancement of γ-aminobutyric acid (GABA) activity, inhibition of GABA degradation, and blockade of voltage-gated sodium, calcium, and potassium channels [1]. In overdose, however, VPA can cause life-threatening complications, including severe central nervous system (CNS) depression, hepatotoxicity, hyperammonemia, and cerebral edema [2]. Although most cases are managed with supportive care, select patients with severe toxicity may benefit from extracorporeal elimination techniques or pharmacological interventions such as L-carnitine [2]. This report describes an unusual case complicated by endoscopically confirmed pharmacobezoar formation and delayed neuromuscular toxicity, features rarely reported in acute VPA overdose and of value to clinicians managing severe poisoning. In addition to describing this case, we provide a review of previously published reports, which are summarized in the discussion.

## Case presentation

A 42-year-old man with bipolar affective disorder and hypertension presented to the emergency department (ED) 90 minutes after an intentional overdose of extended-release VPA totaling 60 g (120 x 500 mg tablets) with co-ingestion of zolpidem 110 mg and paracetamol 2 g. He had been discharged the previous day following a 14-day psychiatric admission without suicidal ideation. He was found drowsy on the floor surrounded by empty medication bottles; earlier that day, he had received an unspecified intramuscular injection for back pain. This was his first known overdose attempt with no history of substance misuse or prior suicidal attempts.

On arrival, he was hypotensive with a blood pressure of 85/55 mmHg and a Glasgow Coma Scale (GCS) score of 10 (eye opening: 2, verbal response: 5, motor response: 3) [3]. Oxygen saturation was initially normal but dropped during brain computed tomography (CT) imaging, and with snoring respirations, he was immediately intubated and started on mechanical ventilation for depressed consciousness and persistent hypoxia. The remainder of the examination was unremarkable. Based on the timing of ingestion, dose, elevated serum valproate concentration, and clinical manifestation consistent with known toxic effects of valproic acid, a probable causal association was established between the overdose and the patient's presentation.

Laboratory results, as summarized in Table 1, showed VPA of >450 µg/mL, ammonia of 76 µmol/L, salicylate of <0.3 mg/dL, and acetaminophen level of <5 µg/mL. Toxicology was consulted immediately, and post-intubation gastrointestinal (GI) decontamination was initiated with 50 g of activated charcoal followed by multiple-dose activated charcoal (MDAC) every four hours. He received one dose of naloxone 0.4 mg intravenous (IV) and L-carnitine (100 mg/kg IV loading dose over 30 minutes, then a maintenance dose of 50 mg/kg every eight hours), which was discontinued the following day.

**Table 1:** Pertinent laboratory findings on day 1 of admission and blood gas results Note: Reference ranges based on Rashid Hospital Laboratory Standards. Admission laboratory results are shown; serial valproic acid and ammonia levels are depicted in Figure 3. Other laboratory parameters remained within reference ranges throughout admission. Blood gas results for the first three days (days 0-2) represent the first reading of each day; blood gas measurements from the remaining days were within normal limits throughout admission. pCo_2_: partial pressure of carbon dioxide, HCO_3_: bicarbonate

Test	Result	Reference range
Valproic acid	>450 µg/mL	50-100 µg/mL
Ammonia	76 µmol/L	16-60 µmol/L
Acetaminophen	<5 µg/mL	10-30 µg/mL
Salicylate	<0.3 mg/dL	15-30 mg/dL
Bilirubin	0.36 mg/dL	0-1.2 mg/dL
Alkaline phosphatase	47 U/L	40-129 U/L
Alanine transaminase	36 U/L	0-41 U/L
Total protein	6.8 g/dL	6.6-8.7 g/dL
Albumin	3.7 g/dL	4.4-5.1 g/dL
Globulin	3.1 g/dL	2.8-3.4 g/dL
Sodium	137 mmol/L	136-145 mmol/L
Potassium	3.7 mmol/L	3.4-4.5 mmol/L
Chloride	99 mmol/L	98-108 mmol/L
Bicarbonate	23 mmol/L	20-28 mmol/L
Urea	29 mg/dL	12-40 mg/dL
Anion gap	15 mmol/L	6-14 mmol/L
Creatinine	0.83 mg/dL	0.70-1.20 mg/dL
Estimated glomerular filtration rate	112.1 mL/min/1.73 m^2^	>60 mL/min/1.73 m^2^
Blood gas (day 0)	pH: 7.417	pH: 7.35-7.45
	pCo_2_: 42.5 mmHg	pCo_2_: 35-45 mmHg
	HCO_3_: 27.3 mmol/L	HCO_3_: 21-28 mmol/L
	Lactate: 1 mmol/L	Lactate: 0.5-1.6 mmol/L
Blood gas (day 1)	pH: 7.397	pH: 7.35-7.45
	pCo_2_: 38.1 mmHg	pCo_2_: 35-45 mmHg
	HCO_3_: 23.3 mmol/L	HCO_3_: 21-28 mmol/L
	Lactate: 0.9 mmol/L	Lactate: 0.5-1.6 mmol/L
Blood gas (Day 2)	pH: 7.29	pH: 7.35-7.45
	pCo_2_: 52.1 mmHg	pCo_2_: 35-45 mmHg
	HCO_3_: 22.6 mmol/L	HCO_3_: 21-28 mmol/L
	Lactate: 0.8 mmol/L	Lactate: 0.5-1.6 mmol/L

**Figure 1:**
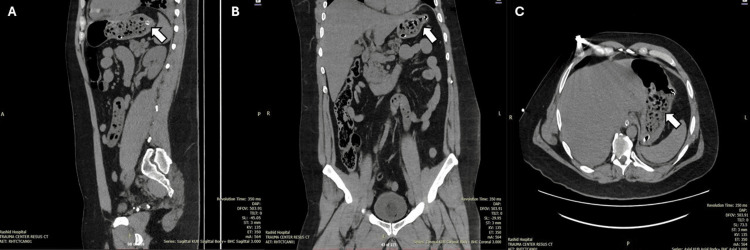
CT of the abdomen and pelvis showing radiolucent tablets consistent with a pharmacobezoar (A-C) Non-contrast CT of the abdomen and pelvis showing multiple small, radiolucent foreign bodies (tablets) seen in the stomach, mainly in the fundus region extending to the pylorus and scattered throughout the small bowel and ascending colon without obstruction. CT: computed tomography

Chest radiograph revealed bilateral basal pneumonitis and pleural effusions. The brain CT was unremarkable. Abdominal radiograph showed no radio-opaque foreign bodies; however, a non-contrast CT of the abdomen/pelvis revealed multiple small, radiolucent foreign bodies within the stomach (primarily fundus/pylorus), with similar material scattered throughout the small bowel and ascending colon without bowel obstruction or perforation (Figure 1).

**Figure 2:**
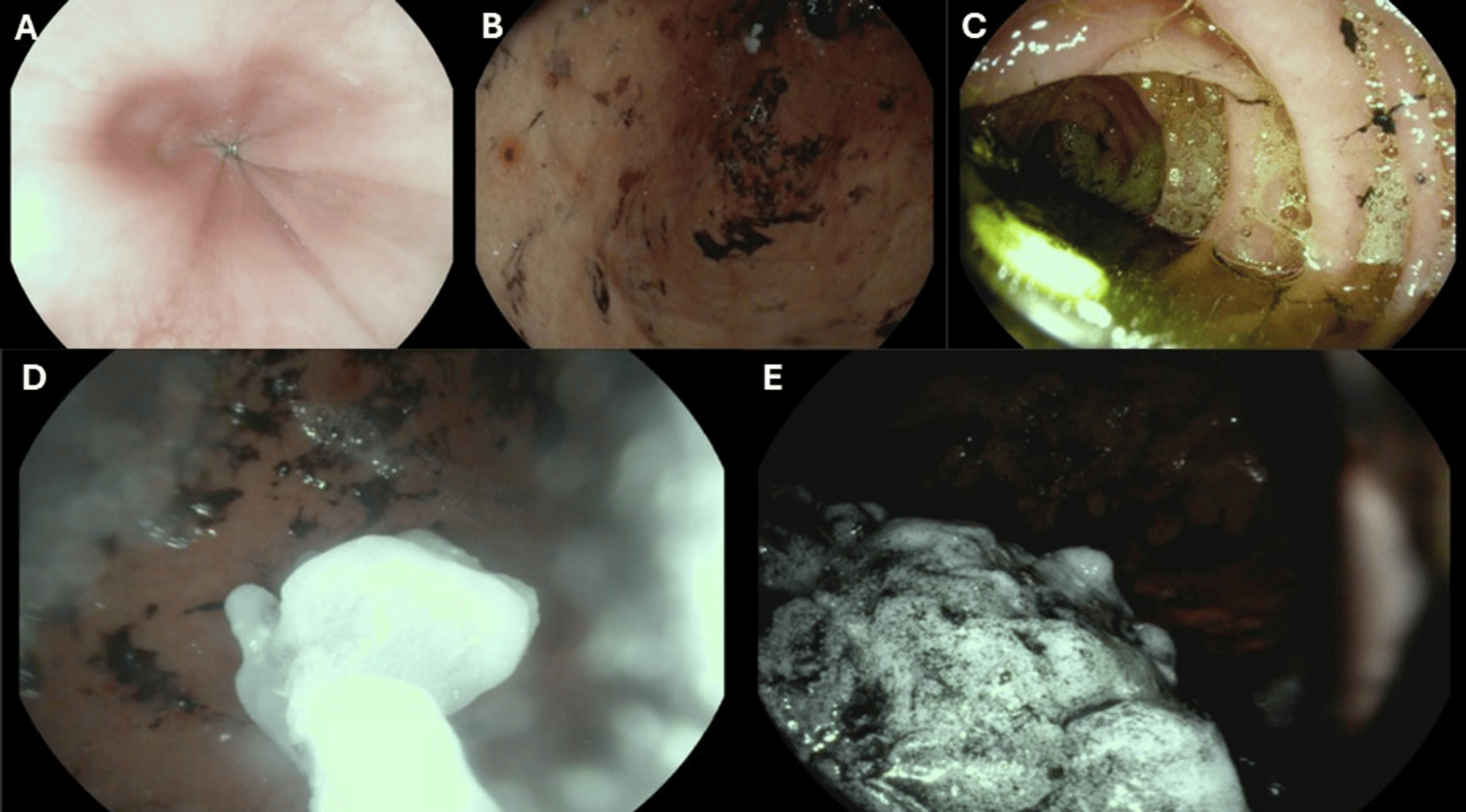
Endoscopic confirmation of pharmacobezoar following valproic acid overdose Upper endoscopy images showing pharmacobezoar formation in the stomach following massive valproic acid ingestion. (A–E) Images demonstrate large clumped VPA tablets occupying the gastric fundus and pylorus. The gastric mucosa is stained with charcoal; tablet fragments are visible throughout. Images courtesy of Dr. Ali Emad Alzaidy, Department of Gastroenterology, Rashid Hospital. VPA: valproic acid

The gastroenterology team was consulted, and upper endoscopy confirmed a large 30-cm clumped mass of VPA tablets, confirming a pharmacobezoar; gastric mucosa was charcoal-stained, and the duodenum appeared diffusely erythematous, with fragmented tablet material present (Figure 2).

**Figure 3:**
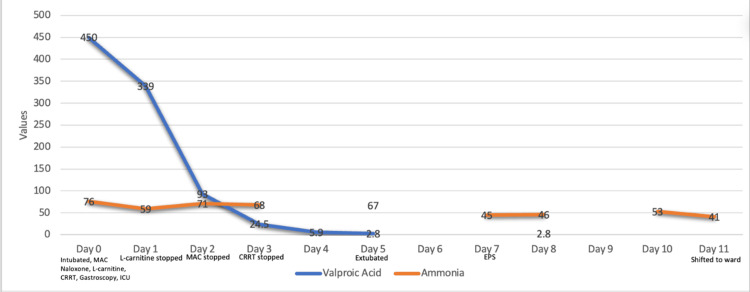
Trend of serum VPA and ammonia levels during hospital stay Note: Multiple serum VPA and ammonia levels were obtained daily; only the initial value for each day is represented in this figure for clarity. VPA: valproic acid, MDAC: multiple-dose activated charcoal, L-carnitine: levocarnitine, CRRT: continuous renal replacement therapy, EPS: extrapyramidal symptoms, ICU: intensive care unit

He was admitted to the intensive care unit (ICU) for continuous renal replacement therapy (CRRT) and serial monitoring of VPA and ammonia levels. Over 72 hours, VPA decreased to <2.8 µg/mL and ammonia gradually normalized (Figure 3). Liver function tests (LFT) and urea/electrolytes (U&E) remained unremarkable. He was extubated on day 5 and transferred to the ward on day 11 in stable condition.

Throughout admission, the patient was co-managed by intensive care, toxicology, psychiatry, internal medicine, and neurology teams. Neurologic complications evolved during admission. He developed bilateral upper and lower limb weakness, rigidity, tremor, rotatory nystagmus, and clonus. The electroencephalogram (EEG) was normal; initial nerve conduction was unremarkable, but repeat testing showed ulnar sensory neuropathy. Electromyography (EMG) of the vastus medialis and deltoid demonstrated myopathic features with profound active denervation. Magnetic resonance imaging (MRI) of the brain with contrast was normal. A diagnosis of toxic myopathy with EPS was made, and procyclidine was initiated. He was transferred to inpatient rehabilitation at another facility and discharged ambulatory after 54 days.

## Discussion

VPA is a broad-spectrum antiepileptic and mood-stabilizing drug [1]. In overdose, it disrupts mitochondrial metabolism, depleting acetyl coenzyme A (CoA) and carnitine stores, and shifts fatty acid metabolism from β-oxidation to ω-oxidation. This leads to the accumulation of toxic metabolites such as 2-propyl-4-pentenoic acid (4-en-VPA), which impair hepatic function and inhibit carbamoyl phosphate synthetase I (CPS-I), contributing to complications including CNS depression, hyperammonemia, and hepatotoxicity [4].

Management strategies are guided by consensus recommendations. The 2008 American Association of Poison Control Centers (AAPCC) expert consensus guideline recommends referral from poison centers to the ED for patients with suicidal intent, symptomatic ingestions, or asymptomatic ingestions > 50 mg/kg; it discourages ipecac, supports activated charcoal within the preceding hour, and considers naloxone for patients with respiratory depression [5]. Our patient received airway protection, GI decontamination, and L-carnitine consistent with these recommendations. Although extracorporeal elimination was not addressed in the 2008 AAPCC guideline, its role has been strongly supported by the Extracorporeal Treatment (EXTRIP) Workgroup (2015), which provides structured recommendations for initiating renal replacement therapy in severe VPA poisoning [6]. These include VPA concentration > 1,300 mg/L, cerebral edema, or shock. Extracorporeal treatment (ECTR) is also suggested if the VPA concentration exceeds 900 mg/L or if the patient has coma, respiratory depression requiring mechanical ventilation, acute hyperammonemia, or pH ≤ 7.10 [6]. Our patient fulfilled EXTRIP criteria with CNS depression and ventilatory support and underwent 72 hours of CRRT with subsequent clinical improvement and a reduction in VPA levels.

Literature from Al Aly et al. [7], Tichelbäcker et al. [8], and Thanacoody [9] further reinforces the use of extracorporeal therapies such as CRRT or hemodialysis (HD) in cases with elevated VPA levels > 850 mg/L or clinical features of severe VPA poisoning such as coma, hemodynamic instability, severe hyperammonemia, and acid-base disturbances. Our patient's presentation mirrored many of the previously published severe VPA overdose cases: CNS depression, hypotension, hyperammonemia, and a VPA level > 450 µg/mL. His treatment included early risk assessment, airway protection, MDAC, L-carnitine, and CRRT, which was consistent with existing literature, including approaches described by Chan et al. [10] and Al Jawder et al. [11]. Al Jawder et al. similarly documented MDAC in a patient who received four 50 g doses of charcoal with a good clinical response [11].

Our case adds two unique findings to the literature. First, while delayed clearance is often described in sustained-release VPA ingestions, limited case reports have confirmed bezoar formation endoscopically [12,13]. In our patient, a 30-cm bezoar of fragmented tablets was directly visualized, confirming a significant source of ongoing absorption and delayed clearance. In a study by Ingels et al., 26 patients with initial VPA levels < 100 µg/mL went on to develop significant toxicity, with some peaking more than eight hours later [14]. This supports the need for serial monitoring and imaging in suspected massive ingestion of sustained-release formulations.

Second, the patient developed delayed neuromuscular toxicity with extrapyramidal features. Bilateral upper and lower limb weakness, tremor, rigidity, clonus, and nystagmus evolved during admission, and EMG confirmed toxic myopathy with denervation. While neuromuscular complications such as myopathy and parkinsonism are documented in chronic VPA therapy, they are rare in the context of acute overdose and were attributed to low carnitine levels [15,16]. Mittal et al. reported myopathy within days of initiating sodium valproate as a therapeutic measure for epilepsy, even in the absence of overdose, reinforcing the hypothesis that carnitine deficiency is a contributing factor [17].

The neurologic picture prompted consideration of critical illness myopathy, extrapyramidal side effects, functional neurologic disorder, and demyelination. EMG findings, clinical timing, and absence of confounding medications supported VPA-induced toxic myopathy.

VPA depletes carnitine stores through various mechanisms, including excretion of valproylcarnitine, reduction of endogenous carnitine synthesis, and inhibition of the membrane carnitine transporter. The resulting mitochondrial dysfunction reduces β-oxidation and impairs the urea cycle, leading to hyperammonemia. L-carnitine is thought to decrease ammonia levels by restoring the β-oxidation process [18]. A Quantitative Systems Pharmacology (QSP) model by Schiavo et al. showed that even when started eight hours after ingestion, a regimen of 6 g of L-carnitine followed by 1 g every four hours effectively reduced high ammonia levels [19]. A randomized controlled trial by Țincu et al. similarly showed improved clinical outcomes and recovery with L-carnitine [20]. However, in a murine model, Jamshidzadeh et al. found that while carnitine reduced oxidative stress, it did not prevent liver injury or encephalopathy [21]. Pagali et al. reported hyperammonemia rebound after discontinuation of L-carnitine, supporting continued therapy for at least 72 hours [22]. Although high-flux HD is more efficient, CRRT can still be used as an alternative and has been used successfully in many case reports [6,11,12,23]. HD has been shown to reduce VPA half-life from approximately 30 hours to 2-3 hours in optimal settings [9].

While not used in our case, carbapenems such as meropenem have been used off-label to enhance VPA clearance by inhibiting acylpeptide hydrolase [24]. Al-Quteimat et al. reported rapid VPA reductions but also noted prolonged suppression and seizure recurrence in over 50% of patients, so they advised the use of an antiepileptic drug as an adjunct with carbapenem [25].

Representative case reports are summarized in Table 2. Some case reports described delayed peak levels but did not confirm the presence of bezoars [14].

**Table 2:** Literature review Summarized reports of severe valproic acid toxicity, highlighting patient characteristics, ingested dose, formulation, serum levels, interventions, and outcomes. This table is intended to provide representative clinical data and is not an exhaustive review of all published cases. Note: Among all reviewed cases, only one explicitly documented bezoar formation, which was confirmed via endoscopy in the case reported by Wang et al. [12]. ND: not documented, ER: extended-release, DR: delayed-release, CRRT: continuous renal replacement therapy, L-carnitine: levocarnitine, HDF: hemodiafiltration, GL: gastric lavage, AC: activated charcoal, MAC: multi-dose activated charcoal, HD: hemodialysis, NAC: N-acetylcysteine, SR: sustained-release, WBI: whole bowel irrigation, CVVHDF: continuous veno-venous hemodiafiltration, HP: hemoperfusion, ECMO: extracorporeal membrane oxygenation, CVVH: continuous veno-venous hemofiltration, FPSA-CVVH: fractionated plasma separation and adsorption - continuous veno-venous hemofiltration, y.o.: year old, F: female, M: male

Author/year	Number of patients	Age/gender	Ingested dose (g)	Formulation	Highest VPA level	Treatment offered/outcomes
Ishikura et al. (1996) [26]	1	16-month-old/M	4 g	ND	1,316.2 μg/mL	GL, AC, IV sodium bicarbonate, L-carnitine/survived
Kane et al. (2000) [27]	1	25 y.o./F	ND	ND	1,294 μg/mL	GL, AC, sodium bicarbonate, high-flux HD/survived
Spiller et al. (2000) [28]	133	Range from 2 to 66 y.o./M and F	ND	ND	16 patients > 850 μg/mL with 2 patients > 1,200 μg/mL	HD (7 patients), L-carnitine (4 patients)/131 survived, 2 died > 1,200 μg/mL
Kroll and Nand (2002) [29]	1	38 y.o./M	45 g	Enteric-coated DR	726 μg/mL	GL, AC, HD/survived
Roberge and Francis (2002) [30]	1	44 y.o./F	ND	ND	138 ug/mL	Naloxone/survived
Ingels et al. (2002) [14]	173 (26 with delayed peak levels)	Of the 26 patients, ranging from 11 to 60, with undocumented gender	ND	23 took divalproex sodium DR (others ND)	1 patient > 800	Varied; most received AC, some underwent GL, WBI, or dialysis/survived
Kay et al. (2003) [31]	1	48 y.o./F	48 g	ND	1,402 μg/mL	GL, osmotic catharsis, AC, IV bicarbonate, CVVHDF, low-flux HD/survived
Kielstein et al. (2003) [32]	1	24 y.o./M	30 g	ND	670 µg/mL	GL, AC, high-flux HD/survived
Meek et al. (2004) [33]	Case 1	38 y.o./M	60 tablets	Depakine Chrono ER	846 μg/mL	AC, magnesium sulfate, IV L-carnitine, HD/survived
Meek et al. (2004) [33]	Case 2	37 y.o./M	ND	Depakine Chrono ER	260 μg/mL	AC/survived
Al Aly et al. (2005) [7]	1	35 y.o./M	30 g	ND	573 µg/mL	Single and MAC, HD + HP, CVVHDF/survived
Wilimowska et al. (2006) [34]	Case 1	16 y.o./F	18 g	Depakine Chrono 500 SR	458 mg/L	GL, AC/survived
Wilimowska et al. (2006) [34]	Case 2	38 y.o./F	22 g	Depakine Chrono 500 SR	724 mg/L	Symptomatic only/survived
Wilimowska et al. (2006) [34]	Case 3	21 y.o./F	30 g	Depakine Chrono 500 SR	517 mg/L	Symptomatic only/survived
Wilimowska et al. (2006) [34]	Case 4	22 y.o./M	15 g	Depakine Chrono 500 SR	461 mg/L	Symptomatic only/survived
Wilimowska et al. (2006) [34]	Case 5	48 y.o./M	19.5 g	Depakine Chrono 500 SR	503 mg/L	Symptomatic only/survived
Katiyar and Aaron (2007) [4]	1	28 y.o./M	ND	Divalproex 500 (ND)	1016 μg/mL	MAC, WBI, L-carnitine/survived
Unal et al. (2007) [35]	1	26-day-old/F	300 mg	ND	268.6 μg/mL	MAC, lactulose, naloxone, L-carnitine/died
Chan et al. (2007) [10]	Case 1	14 y.o./F	20 g	SR (Epilim Chrono)	288 mg/L	AC, naloxone, L-carnitine/survived
Chan et al. (2007) [10]	Case 2	19 y.o./M	ND	SR (Epilim Chrono)	950 mg/L	MAC, L-carnitine/survived
Mestrović et al. (2008) [36]	1	16 y.o./F	75 g	Enteric-coated DR	1,320 μg/mL	Naloxone, HD/survived
Sikma et al. (2008) [37]	1	41 y.o./M	Estimated >100 g	ND	1,308 μg/mL	MAC, L-carnitine/survived
Jung et al. (2008) [38]	1	23 y.o./F	24 g	ND	1,159.4 μg/mL	GL, MAC, HP, IV L-carnitine, naloxone, lactulose/survived
Jacobs et al. (2008) [39]	1	54 y.o./F	ND	ND	1,664 μg/mL	CVVHDF and supportive/ND
van der Wouden et al. (2009) [13]	1	57 y.o./M	64 g	Depakine 300 enteric-coated + Depakine Chrono 500 ER	1,069 μg/mL	AC, GL, HD, CVVH-D/survived
Waring and Nixon (2009) [40]	1	36 y.o./F	32 g	Epilim (likely immediate-release)	630 μg/mL	Supportive/survived
Leitch and Williams (2010) [41]	1	41 y.o./F	ND	ND	534 mg/L	Naloxone, carnitine/survived
Crudup et al. (2011) [42]	1	30 y.o./M	ND	ND	990 µg/mL	Supportive and HD/survived
Nasa et al. (2011) [43]	1	21 y.o./M	30 g	Enteric-coated DR tablets	1,060 µg/mL	GL, naloxone, AC, high-flux HD, L-carnitine/survived
Pons et al. (2012) [44]	1	32 y.o./F	25 g	ND	1,160 mg/L	HD/survived
Atmaca Temrel et al. (2013) [45]	1	31 y.o./F	ND	SR	>150 μg/mL	GL, HD, L-carnitine, MAC/survived
Ray and Skellett (2013) [46]	1	2 y.o./F	4 g	Non-modified release	611 mg/L	CVVH/survived
Schrettl et al. (2017) [47]	Case 1	40 y.o./M	18 g	DR	542 mg/L	AC, HD, L-carnitine, L-arginine/survived
Schrettl et al. (2017) [47]	Case 2	27 y.o./M	30 g	ND	439 mg/L	MAC L-carnitine, HD, L-arginine/survived
Schrettl et al. (2017) [47]	Case 3	32 y.o./F	56 g	DR	691 mg/L	L-arginine, HD/survived
Schrettl et al. (2017) [47]	Case 4	41 y.o./F	27 g	DR	484 mg/L	AC, L-arginine/survived
Schrettl et al. (2017) [47]	Case 5	38 y.o./F	30 g	DR	749 mg/L	AC, L-arginine/survived
Muñiz (2017) [48]	1	16 y.o./F	5 g	ND	294 μg/mL	AC/survived
Ge et al. (2017) [49]	1	33 y.o./F	20 g	ND	420.84 mg/L	GL, magnesium sulfate catharsis, MAC, polyene phosphatidylcholine, L-carnitine, nalmefene infusion, FPSA-CVVH/survived
Khobrani et al. (2018) [50]	1	45 y.o./M	10 g	ER divalproex sodium	415 μg/mL	Naloxone, AC, L-carnitine, meropenem/survived
Tichelbäcker et al. (2018) [8]	1	46 y.o./F	56 g	ND	>10,389.5 µmol/L	L-carnitine, HDF/survived
Al Jawder et al. (2018) [11]	1	51 y.o./F	60 g	ND	905 µg/mL	Single and MAC, L-carnitine, CRRT, HD/survived
Mekonnen (2019) [51]	1	4 y.o./F	ND	ND	681 mg/L	L-carnitine/survived
Dreucean et al. (2019) [52]	1	38 y.o./F	Not documented (estimated 20 tablets of extended-release divalproex sodium)	ER divalproex sodium	278 μg/mL	AC, L-carnitine, meropenem/survived
Özer et al. (2019) [53]	1	34 y.o./F	45 g	ND	386 μg/mL	GL, AC, L-carnitine/survived
Thomas et al. (2020) [54]	1	42 y.o./F	26 g	ER (divalproex sodium ER)	224 μg/mL	Naloxone, AC, ertapenem, meropenem/survived
Sanivarapu et al. (2021) [55]	1	42 y.o./F	ND	ER	144 µg/mL	AC, carnitine, meropenem/survived
Patel et al. (2022) [56]	1	56 y.o./M	6 g	ER (divalproex sodium)	275.1 µg/mL	L-carnitine/survived
Comstock et al. (2022) [57]	1	17 y.o./F	44 g	ND	2,226 mg/L	AC, L-carnitine, high-dose CVVHDF/survived
Cunningham et al. (2022) [58]	1	38 y.o./F	ND	DR divalproex sodium	243.6 μg/mL	L-carnitine, meropenem/survived
Doar et al. (2023) [59]	1	47 y.o./M	30 g	DR (Depakote DR 500 mg)	>300	L-carnitine, HD/survived
Perković Vukčević et al. (2024) [24]	Case 1	60 y.o./F	30 g	ER	265 mg/L	AC, meropenem/survived
Perković Vukčević et al. (2024) [24]	Case 2	39 y.o./M	ND	ND	344.18 mg/L	AC, meropenem, HP, lipid emulsion/died
Perković Vukčević et al. (2024) [24]	Case 3	61 y.o./F	45 g	ND	496 mg/L	Meropenem/survived
Perković Vukčević et al. (2024) [24]	Case 4	22 y.o./M	30 g	ND	480 mg/L	Meropenem/survived
Perković Vukčević et al. (2024) [24]	Case 5	58 y.o./F	30 g	ND	269.60 mg/L	Meropenem/survived
Perković Vukčević et al. (2024) [24]	Case 6	53 y.o./M	7.5 g	ND	114 mg/L	Meropenem/survived
Perković Vukčević et al. (2024) [24]	Case 7	27 y.o./F	30 g	ND	135.8 mg/L	Meropenem/survived
Perković Vukčević et al. (2024) [24]	Case 8	27 y.o./F	ND	ND	724 mg/L	Naloxone, imipenem/survived
Perković Vukčević et al. (2024) [24]	Case 9	42 y.o./M	1.5 g	ND	199.65 mg/L	Meropenem/survived
Perković Vukčević et al. (2024) [24]	Case 10	43 y.o./F	22.5 g	ND	249 mg/L	Meropenem/survived
Perković Vukčević et al. (2024) [24]	Case 11	68 y.o./F	ND	ND	167.70 mg/L	Meropenem/survived
Romero Carratala et al. (2024) [60]	1	59 y.o./F	ND	ND	503.06 μg/mL	AC, naloxone, flumazenil, L-carnitine, meropenem/survived
Hussan et al. (2024) [61]	1	Mid-20s/M	13 g	ND	975 μg/mL	L-carnitine, NAC, CVVHDF, ECMO/survived
Liu et al. (2024) [23]	Case 1	14 y.o./F	30 g	SR magnesium valproate	635.77 mg/L	GL, HD, HP, CRRT, ornithine/survived
Liu et al. (2024) [23]	Case 2	13 y.o./F	13.6 g	ND	507.00 mg/L	GL, HP, heparin, dopamine/survived
Liu et al. (2024) [23]	Case 3	14 y.o./F	10.5 g	SR sodium valproate	317 mg/L	GL, HP, naloxone, furosemide, heparin, dopamine/survived
de Bairros et al. (2025) [62]	1	15 y.o./F	Unclear	ND	977.96 µg/mL	GL with AC, urinary alkalinization with sodium bicarbonate/died
Wang et al. (2025) [12]	1	16 y.o./F	40 g	ND	265 µg/mL	CRRT, HF, GL/survived

Limitations of this case include the initially negative abdominal radiograph, which did not reveal any radio-opaque foreign bodies or bezoars. This reflects the radiolucency of VPA tablets and highlights the risk of delayed recognition, as plain films may be falsely reassuring in cases of massive ingestion. Given the high clinical suspicion and substantial ingested dose, early toxicology consultation prompted a CT scan of the abdomen and pelvis, which confirmed multiple radiolucent foreign bodies. Additional challenges included the incomplete initial history and reliance on collateral information from family members to guide management.

## Conclusions

This case is distinct in documenting both an endoscopically confirmed pharmacobezoar and delayed neuromuscular toxicity following acute massive VPA overdose, features that remain rarely reported in the literature. Clinicians should maintain a high index of suspicion in massive overdoses, as VPA toxicity may result in prolonged absorption, persistent toxicity, and serious complications requiring aggressive interventions.

Timely diagnosis and early management, including airway management, activated charcoal, L-carnitine, CRRT, and supportive care, were essential to patient survival and contributed to a favorable outcome. Imaging and endoscopy should be considered in sustained-release overdoses or when delayed gastrointestinal clearance is suspected. Finally, this case underscores the vital role of the toxicology service for on-call consultation, risk stratification, and guidance on advanced interventions, as well as the importance of coordinated multidisciplinary care in managing such complex toxicologic emergencies.
